# Atorvastatin does not ameliorate nephrogenic diabetes insipidus induced by lithium or potassium depletion in mice

**DOI:** 10.14814/phy2.15111

**Published:** 2021-11-11

**Authors:** Maria L. Thomsen, Camilla Grønkjær, Anna Iervolino, Soham Rej, Francesco Trepiccione, Birgitte M. Christensen

**Affiliations:** ^1^ Department of Biomedicine Aarhus University Aarhus C Denmark; ^2^ Department of Translational Medical Sciences University of Campania “L. Vanvitelli” Naples Italy; ^3^ Biogem Institute of Molecular Biology and Genetics Ariano Irpino Italy; ^4^ Jewish General Hospital/Lady Davis Institute/Department of Psychiatry McGill University Montreal Quebec Canada

**Keywords:** AQP2, hypokalemia, lithium, NDI, statin

## Abstract

Acquired forms of nephrogenic diabetes insipidus (NDI) include lithium (Li)‐induced and hypokalemia‐induced NDI. Both forms are associated with AQP2 downregulation and collecting duct (CD) cellular remodeling. Statins are cholesterol‐lowering drugs appearing to increase AQP2 membrane‐translocation and improve urine concentration in other NDI models. We have investigated if statins are able to prevent or rescue the Li‐induced changes in mice and in a mouse cortical CD cell line (mCCD_c1l_). Biotinylation assays showed that acute (1hr) atorvastatin, simvastatin, or fluvastatin increased AQP2 membrane accumulation in mCCD_c1l_ cells showing that the cell line responds to acute statin treatment. To see whether chronic statin treatment abolish the Li effects, mCCD_c1l_ cells were treated with 48 h Li, combined Li/atorvastatin or combined Li/simvastatin. Li reduced AQP2, but combined Li/atorvastatin or Li/simvastatin did not prevent AQP2 downregulation. In mice, chronic (21 days) Li increased urine output and reduced urine osmolality, but combined Li/atorvastatin did not prevent these effects. In inner medulla (IM), Li reduced total AQP2 and increased pS261‐AQP2. Combined Li/atorvastatin did not abolish these changes. Atorvastatin did not prevent a Li‐induced increase in intercalated cells and proliferation in IM. In mice with already established NDI, atorvastatin had no effect on the Li‐induced changes either. Mice subjected to 14 days of potassium‐deficient diet developed polyuria and AQP2 downregulation in IM. Co‐treatment with atorvastatin did not prevent this. In conclusion, atorvastatin does not appear to be able to prevent or rescue Li‐NDI or to prevent hypokalemic‐induced NDI.

## Graphical abstract

Patients with nephrogenic diabetes insipidus (NDI) suffer from severe polyuria and polydipsia. Statins have been suggested as potential treatment. However, we found that statins have no effect in two acquired forms of NDI.

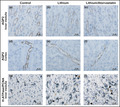

## 1 INTRODUCTION

Nephrogenic diabetes insipidus (NDI) is characterized by the inability of the kidney to concentrate urine in response to vasopressin. NDI can be inherited, but it is in most cases an acquired disease. Acquired NDI can result from for example, drug treatments or electrolyte disorders. Lithium (Li) is a first‐line mood‐stabilizing drug used in the treatment of affective disorders (Boton et al., 1987), but as a side effect it can cause urinary concentrating difficulties in up to 50% of the patients, and induce clinically important NDI in approx. 20% of chronic users (Yatham et al., 2018). We have recently shown that already after 8 weeks of treatment, Li impairs the maximal urinary concentration ability in previously Li‐naïve patients (Sacca et al., 2015). In the most severe form of NDI, patients can produce up to 20 L of urine per day and must have an equally large fluid intake to avoid severe dehydration. Patients subjected to multiple years of Li treatment may develop microcysts and are in risk of developing chronic renal failure (Davis et al., 2018). Beside the use in treating bipolar disorders and depression, Li has also been mentioned as a potential drug in Alzheimer disease, stroke, and other neuropsychiatric conditions (Matsunaga et al., 2015).

Li enters the kidney collecting duct (CD) principal cells through the epithelial sodium channel (ENaC; Christensen et al., 2011; Kortenoeven et al., 2009). Li interrupts the AVP pathway and decreases cAMP‐production and PKA‐activity, which reduces AQP2 expression and membrane accumulation (Trepiccione & Christensen, 2010). Evidence suggests that Li increases the phosphorylation of ERK1/2, which precedes the increase in the phosphorylation of Ser261 in AQP2, which may be involved in decreased AQP2 levels (Trepiccione et al., 2014). Li also causes a cellular remodeling of the CD, leading to a higher fraction of intercalated cells compared to principal cells and an increased cellular proliferation (Christensen et al., 2004; 2006; Trepiccione et al., 2013) altering not only the water reabsorption ability, but also the acid‐base homeostasis of the CD (Trepiccione et al., 2018).

The current treatment for Li‐NDI is thiazide diuretics, amiloride, and low‐salt diet, which are not completely effective (Kirchlechner et al., 1999). Thus, an effective treatment of Li‐NDI is still lacking. Statins have been used for dyslipidemia for many years, and their effect is exerted by inhibiting the activity of 3‐hydroxy‐3‐methyl‐glutaryl‐CoA reductase (HMG‐CoA reductase), which will result in decreased biosynthesis of cholesterol (Sirtori, 2014). Besides their cholesterol‐lowering effect, statins also have a variety of pleiotropic effects, including stabilizing atherosclerotic plaque, reducing vascular inflammation, and improving endothelial function (Bedi et al., 2016). Recent studies have indicated that statins could have an effect on AQP2 membrane accumulation. Simvastatin has been shown to improve urine concentration ability and to increase AQP2 surface expression in Brattleboro (BB) rats (Li et al., 2011), a model of central DI. In mice with X‐linked NDI, fluvastatin in combination with secretin increased AQP2 membrane expression as well as the urine concentration ability (Procino et al., 2011, 2014). Furthermore, in hypercholesterolemic patients receiving simvastatin for 12 weeks, urine output was decreased, and uAQP2 was increased (Procino et al., 2016). In a cross‐sectional study of 71 lithium users, 0% (0/17) of statin users compared with 20.4% (11/54) of statin nonusers had NDI (Elie et al., 2015). The purpose of the current study was to investigate the effect of statins in Li‐NDI. We tested whether atorvastatin or simvastatin could prevent a Li‐induced AQP2 downregulation in a cortical CD cell line, and whether atorvastatin have protective effects in mice with Li‐NDI. We furthermore tested the effect of atorvastatin in another acquired form of NDI, that is, in hypokalemic rats. Potassium deficiency is known to result in polyuria, polydipsia, AQP2 downregulation, and cellular remodelling (Iervolino et al., 2020; Marples et al., 1996). This is associated with increased autophagic degradation of AQP2 (Khositseth et al., 2015; Kim et al., 2019). Whether statins have an effect in hypokalemic‐induced NDI is not known.

## 2 MATERIALS AND METHODS

### 2.1 Drugs and reagents

Lithium, atorvastatin, fluvastatin, and simvastatin were purchased from Sigma‐Aldrich. Atorvastatin was also purchased from Merck Life Science (prevention and potassium‐deficient [KD] animal experiment). The atorvastatin was dissolved in dimethyl sulfoxide forming a 10 mg/ml stock solution. Simvastatin, which is a prodrug, was diluted in ethanol followed by activation in NaOH and then heating for 2 h at 50°C. The solution was neutralized with HCl and adjusted with phosphate buffered saline (PBS) to a stock solution of 10 mg/ml.

### 2.2 Cell culture

Mouse cortical CD cell line cells (Gaeggeler et al., 2005, 2011) (passage 28–36) were seeded with 380,000 cells per filter (Transwell, 0.4μm polyester membrane; Corning Costar). They were grown for 7 days until confluence at 37°C. A transepithelial electrical resistance ≥1000 mΩ was required before initiation of experiments. Throughout the experiments, cells were kept in Dulbecco's Modified Eagle's Medium F:12 from Gibco with 2% fetal calf serum. The medium was supplemented with a 1% penicillin‐streptomycin solution as well as insulin, transferrine, Na‐selenate, dexametasone, triiodo‐L‐thyronine, and epidermal growth factor. In all experiments, cells were treated with basolateral [deamino‐Cys^1^‐D‐Arg^8^]‐vasopressin (dDAVP) (1 nM) for 4 days (Kortenoeven et al., 2009). For long‐term (48 h) experiments, cells were treated for the last 48 h with +/− lithium (apical, 10 mM; Kortenoeven et al., 2009), +/− atorvastatin (1 μM, basolateral), and +/− simvastatin (15 μM, apical/basolateral; Chen et al., 2014; Hamasaki et al., 2012; Procino et al., 2010). When biotinylation was performed, atorvastatin was administered from both the apical and basolateral side. For short‐term (1 h) experiments, cells were subjected to wash‐out in pure media for 2 h followed by treatment for 1 h with either dDAVP (1 nM, basolateral), atorvastatin (40, 80 and 120 μM, apical/basolateral), fluvastatin (100 μM, apical/basolateral), or simvastatin (150 μM, apical/basolateral; Li et al., 2011; Procino et al., 2011). After experiments, filters were rinsed in 0.01 M PBS and treated with protease‐ and phosphatase‐inhibitors. Samples were subsequently sonicated and centrifuged for 10 min at 4°C. 1× sample buffer with dithiothreitol (DTT) was added to the supernatant, and samples were incubated at 65°C for 10 min. All experiments were performed three to four times and the blotting data from the individual experiments within a given protocol was pooled.

### 2.3 Surface biotinylation

To quantify membrane proteins, cell surface biotinylation assay was performed in accordance with Moeller et al. (Moeller et al., 2016). Proteins were biotinylated by applying the cell‐impermeable Sulfo‐NHS‐SS‐Biotin (Thermo scientific). For the long‐term experiments, EZ‐Link Hydrazide Biocytin (Thermo scientific) was also added and cells were incubated with Na‐meta‐periodate (20 mM) in coupling buffer for 30 min. Cells were washed in coupling buffer and then biotin (1 mg/ml) +/−BiocytinHydrazide (2,5 mM) was added to the apical surface for 30 min at 4°C. Cells were incubated with Quenching Solution for 5 min and scraped off in lysis buffer following sonication and centrifugation for 20 min. The 50 μl supernatant was collected to achieve a total protein sample. To obtain the biotinylated protein sample, the remaining supernatant was transferred to a NeutrAvidin column and columns were incubated for 1 h at room temperature while mixing and then washed in PBS with protease‐ and phosphatase‐inhibitors. Sample buffer with DTT was added to the eluate and were incubated for 1h at room temperature followed by heating for 10 min at 95°C.

### 2.4 Experimental animals

All animal experiments were approved by the Animal Experiments Inspectorate (2013‐15‐2934‐00957 and 2020‐15‐0201‐00625). Male C57BL/6 mice weighing 19–25 g were obtained from Taconic Biosciences (used for lithium studies) and from Janvier Labs, Le Genest‐Saint‐Isle, France (used for potassium depletion study). All mice had free access to standard chow and water.

**FIGURE 1:**
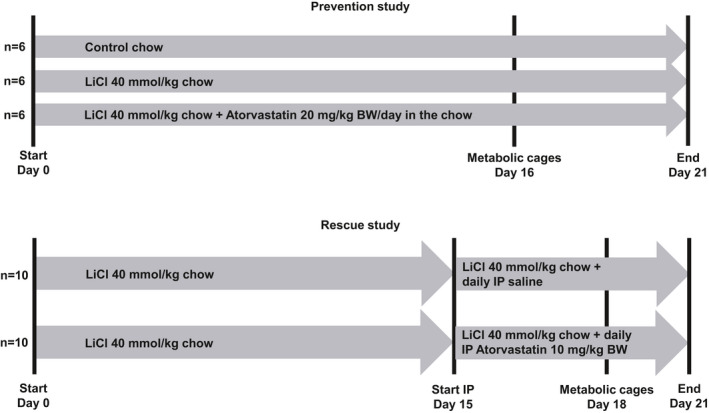
Protocols for prevention and rescue animal experiments. Diagram showing the protocols and the number of animals used in the two animal experiments (prevention and rescue study)

In the prevention experiment (Figure 1, upper panel), 18 mice were divided into three groups (*n* = 6 in each group); (1) normal chow; (2) lithium‐enriched chow (40 mmol LiCl/kg food); (3) lithium (40 mmol LiCl/kg food)/atorvastatin (~20 mg/kg BW/day)‐enriched chow. Food was given ad libitum for 21 days. Mice were monitored in metabolic cages the last 5 days of the experiment.

In the rescue experiment (Figure 1, lower panel), 20 mice received lithium‐enriched chow (40 mmol LiCl/kg food) for 21 days. Atorvastatin (10 mg/kg BW) or saline was administered by daily intraperitoneal injection during the last 6 days of the experiment (*n* = 10 in each group). Mice were monitored in metabolic cages during the last 3 days of the experiment.

At the end of the experiments, mice were anesthetized with isoflurane and one kidney was removed for immunoblotting. The mice were then perfusion fixed via the left ventricle with 4% PFA in PBS and the other kidney was removed for immunohistochemistry. In the prevention experiment, a blood sample was taken from the right atrium before fixation. The prevention experiment was started over two consecutive days (*n* = 3 mice in each group the first day and remaining *n* = 3 mice in each group the second day) and fixation and blood sampling was similarly performed over two consecutive days. The rescue experiment was also started over two consecutive days (*n* = 5 mice in each group the first day and the remaining *n* = 5 mice in each group the second day) and termination of the experiment was also performed over two consecutive days. Mice were perfusion fixed one of the days (*n* = 5 in each group).

To study the impact of atorvastatin in mice subjected to a KD diet (Altromin Spezialfutter GmbH & Co, Germany), 18 mice were randomly divided into three experimental groups; Standard diet (*n* = 6), KD diet (*n* = 6), and KD diet +20 mg/kg BW atorvastatin (*n* = 6, Figure 9 upper panel). The animals were kept on the diet for 14 days. The last 3 days of the study, the mice were kept in metabolic cages. After 14 days, mice were anesthetized with isoflurane, kidneys were extracted and blood was collected via cardiac puncture.

### 2.5 Immunoblotting

Samples from the cell and the animal experiments were subjected to Polyacrylamide gel electrophoresis using Criterion precast 4%–15% gels. Proteins were blotted onto PVDF membranes using the Trans‐Blot^®^ Turbo Transfer System (BioRad). Membranes were blocked in 5% skimmed milk in PBS‐T at room temperature for 1 h followed by washing in PBS‐T and incubation overnight with the primary antibody ([AQP2, 7661AP, dilution 1:1000 or 1:2000; Nielsen et al., 2006], [proteasome 20S, dilution 1:1000; Santa Cruz], [pS261‐AQP2, dilution 1:1000; Hoffert et al., 2007]; [H^+^‐ATPase, H7659AP, dilution 1:2000; Christensen et al., 2006]). Detection of bands was achieved by incubating 1 h with goat‐anti‐rabbit horseradish peroxidase‐conjugated secondary antibody (P448, dilution 1:5000). Images were acquired by ImageQuant LAS 4000 and the density of bands was analyzed by ImageStudio or Quantity One software. In the biotinylation experiments, biotinylated AQP2 was normalized to total AQP2. Furthermore, in the biotinylation experiments, the density of the total AQP2 bands was corrected by the proteasome 20S blots (for presentation of total AQP2 levels). In all other experiments, equal loading on blots was ensured by coomassie staining prior to the blotting. The density of each band was divided by the mean signal of the controls.

### 2.6 Immunohistochemistry

Perfusion‐fixed kidneys were postfixed for 2 h in 4% PFA in PBS at 4°C and embedded in paraffin. For single labeling, sections (2 μm) were blocked with 0.3% H_2_O_2_ in methanol for 30 min followed by antigen retrieval in TEG‐buffer. The sections were treated with 50 mM NH_4_Cl in PBS for 30 min followed by washing with 1% bovine serum albumin (BSA), 0.2% gelatin, 0.05% saponin in PBS. The sections were incubated with AQP2‐antibody (7661 AP, dilution 1:16,000; Nielsen et al., 2006) at 4°C overnight. Next day, sections were rinsed in 0.1% BSA, 0.2% gelatin, 0.05% saponin in PBS and incubated with the goat‐anti‐rabbit horseradish peroxidase‐conjugated secondary antibodies (P448, dilution 1:200; DAKO) for 1 h. Labeling was detected with diaminobenzi‐dinetetrahydrochloride (DAB) and sections were counter stained with Mayers Hematoxylin. For double‐labeling, sections were incubated with mouse‐anti‐proliferating cell nuclear antigen (PCNA) antibodies (dilution 1:10,000, Sigma‐Aldrich) overnight and labeling was detected by goat‐anti‐mouse horseradish peroxidase‐conjugated secondary antibody (P447, dilution 1:200, DAKO) and DAB. This was followed by incubation with rabbit‐anti‐H^+^‐ATPase‐antibodies (dilution 1:200; Christensen et al., 2006) and at day 3, H^+^‐ATPase labeling was visualized with goat‐anti‐rabbit horseradish peroxidase‐conjugated secondary antibody (P448, dilution 1:200, DAKO) and Vector SG (Vector Laboratories). Antigen retrieval was not performed in the double‐labeling procedure.

### 2.7 Urine and blood analysis

Urine osmolality was measured using a freezing point depression osmometer (3320 Micro Osmometer; Advanced Instruments). Serum samples were analyzed at Aarhus University Hospital, Skejby for cholesterol measurement. Total cholesterol, high‐density lipoprotein (HDL), and triglycerides were measured directly and low‐density lipoprotein (LDL) were calculated using the Friedewald formula. Sodium and potassium concentrations were measured by flame photometry (420 Flame Photometer; Sherwood Scientific Ltd).

### 2.8 Statistical analysis

For comparison of more than two groups, data meeting statistical assumptions of normality and variance homogeneity was analyzed by one‐way ANOVA or two‐way ANOVA with Tukey's multiple comparisons. In case of unequal variance, Brown–Forsythe and Welch ANOVA with Dunnet's T3 multiple comparisons were performed. For comparison of only two groups, data meeting statistical assumptions of normality and variance homogeneity was analyzed by an unpaired *t*‐test. In case of unequal variance, data were analyzed by Welch's *t*‐test. Data not meeting assumptions of normality were log‐transformed or square root‐transformed and analyzed by the appropriate tests. If data did not fulfill assumptions of normality after transformation, untransformed data were analyzed by Kruskal–Wallis test (for comparison of more than two groups) or Mann–Whitney test (for comparison of two groups). *p* < 0.05 was considered significant.

## 3 RESULTS

### 3.1 Acute (1 h) atorvastatin, simvastatin, or fluvastatin treatment increases AQP2 sorting to the apical membrane in mCCD_c1l_ cells

**FIGURE 2:**
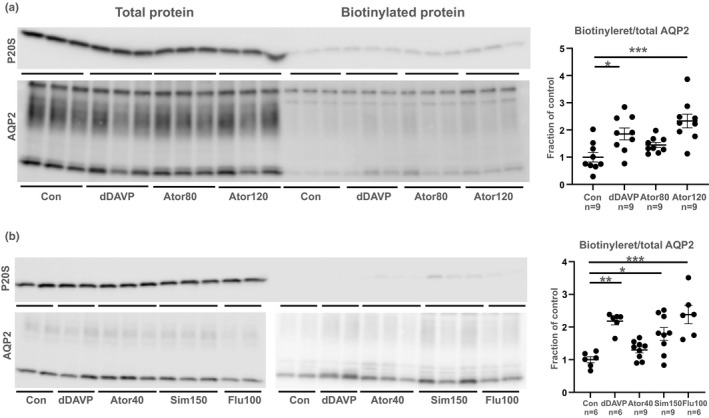
Membrane accumulation of AQP2 after acute (1 h) atorvastatin, simvastatin, or fluvastatin in mCCD_c1l_ cells. Panel (a): Western blot showing the acute (1 h) effect of dDAVP (1 nM) and atorvastatin (80 and 120 μM). Panel (b): Western blot showing the acute (1 h) effect of dDAVP (1 nM), atorvastatin (40 μM), simvastatin (150 μM), and fluvastatin (100 μM) in mCCD_c1l_ cells. Western blot with the intracellular marker proteasome 20S ensured that a minimal amount of intracellular protein was biotinylated (10.7%, 1.9%, and 9.2%, respectively in the three experiments performed in panel (a) and 6.7%, 0.7%, and 2.7%, respectively in the three experiments performed in panel b). Pooled densitometries are shown as mean ± SE. The densitometries are based on both glycosylated and non‐glycosylated AQP2 and are pooled from three identical experiments. All experimental conditions are compared to the control. *p < 0.05; **p < 0.01; ***p < 0.001

We tested the acute effect (1 h) of atorvastatin, simvastatin, and fluvastatin on AQP2 membrane abundance in mCCD_c1l_ cells. Surface biotinylated AQP2 was significantly upregulated in the dDAVP group compared to control confirming vasopressin‐induced AQP2 trafficking (Figure 2a,b). Similarly, atorvastatin (120 μM) significantly increased surface biotinylated AQP2 compared to control at a similar extent as dDAVP (Figure 2a). 80 µM and 40 µM atorvastatin showed a tendency toward upregulation of apical AQP2 expression (Figure 2a,b). Both simvastatin (150 µM) and fluvastatin (100 µM) significantly increased surface biotinylated AQP2 compared to control (Figure 2b). Thus, we can conclude that the mCCD_c1l_ cells respond to acute statin treatment by increasing AQP2 membrane abundance as shown before in other cellular systems using the same doses of statins. Blots were probed with the intracellular marker proteasome 20S, which confirmed biotinylation of membrane proteins and not intracellular proteins (Figure 2a,b).

### 3.2 Atorvastatin does not prevent the lithium‐induced AQP2 downregulation in mCCD_c1l_ cells

**FIGURE 3:**
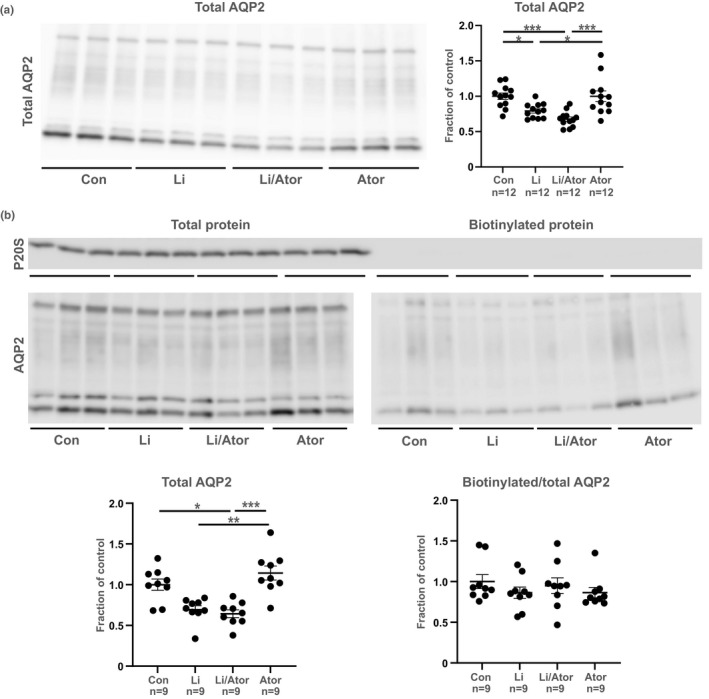
Atorvastatin (48 h) did not prevent the Li‐induced downregulation of total AQP2 in mCCD_c1l_ cells. Panel (a): Western blot showing the effect of Li, Li/atorvastatin and atorvastatin on total AQP2 abundance in mCCD_c1l_ cells. Panel (b): Total AQP2 abundance and fraction of biotinylated AQP2 in mCCD_c1l_ cells treated with Li, Li/atorvastatin and atorvastatin. Western blot with proteasome 20S ensured that a minimal amount of intracellular protein was biotinylated (20.5%, 7.3%, and 0.5%, respectively in the three experiments). Densitometry data are shown as mean ± SE. The densitometry of AQP2 is based on both glycosylated and non‐glycosylated AQP2 and is pooled from three or four identical experiments. *p < 0.05; **p < 0.01; ***p < 0.001

Li treatment for 48 h significantly decreased AQP2 abundance in mCCD_c1l_ cells (Figure 3a). In the biotinylation experiment, there was a tendency to downregulation of total AQP2 in response to Li although not statistically different (Figure 3b, left). Combined Li/atorvastatin treatment for 48 h significantly downregulated AQP2 levels compared to controls and there were no significant changes between the Li and Li/atorvastatin group (Figure 3a,b). Thus, atorvastatin did not prevent the Li‐induced AQP2 downregulation. There were no changes between the control and the atorvastatin group showing that atorvastatin alone does not affect AQP2 abundance (Figure 3a,b). Consistent with these results, AQP2 abundance in the Li group was downregulated compared to the atorvastatin group (Figure 3a,b). Biotinylation assays showed that the membrane abundance of AQP2 was not different between the four groups (Figure 3b, right). Thus, 48 h atorvastatin had no effect on AQP2 trafficking.

### 3.3 Simvastatin (48 h) does not abolish the lithium‐induced AQP2 downregulation and have a negative effect on AQP2 trafficking in mCCD_c1l_ cells

**FIGURE 4:**
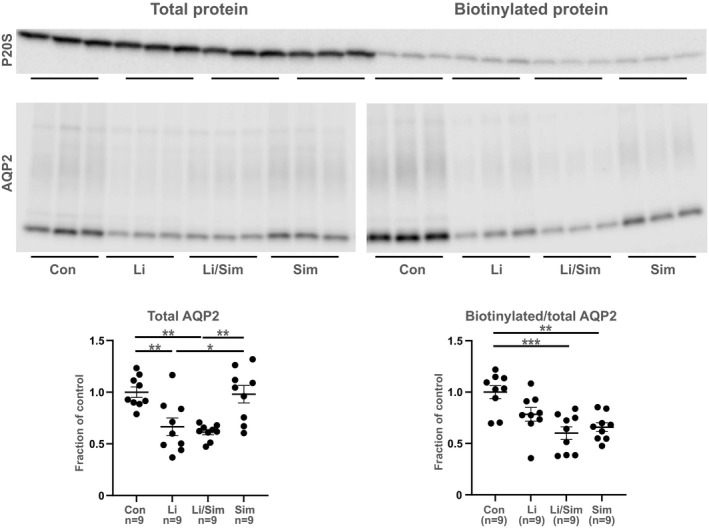
Simvastatin (48 h) did not prevent the Li‐induced downregulation of total AQP2 in mCCD_c1l_ cells and had a negative effect on AQP2 trafficking. Western blotting of the total pool of AQP2 and the fraction of biotinylated AQP2 in response to Li, combined Li/simvastatin and simvastatin in mCCD_c1l_ cells. Western blot with proteasome 20S ensured that a minimal amount of intracellular protein was biotinylated (6.7%, 0.7%, and 2.7%, respectively in the three experiments). Densitometry data are shown as mean ± SE. The densitometry of AQP2 is based on both glycosylated and non‐glycosylated AQP2 and is pooled from three identical experiments. *p < 0.05; **p < 0.01; ***p < 0.001

Both Li and Li/simvastatin treatment significantly decreased total AQP2 levels compared to controls (Figure 4, left). There were no significant changes between the Li and the Li/simvastatin group. Thus, simvastatin did not prevent the Li‐induced AQP2 downregulation. There were no changes between the control and the simvastatin group and consistently the Li group were significantly downregulated compared to the simvastatin group. The levels of surface biotinylated AQP2 were significantly decreased in the Li/simvastatin and the simvastatin group compared to the controls (Figure 4, right). There was a tendency to downregulation in the Li group compared to the control group. No differences between the Li and the Li/simvastatin group were observed. Thus, 48 hrs simvastatin had a negative effect on AQP2 trafficking.

### 3.4 Co‐treatment with atorvastatin and lithium does not prevent lithium‐induced NDI in mice

**FIGURE 5:**
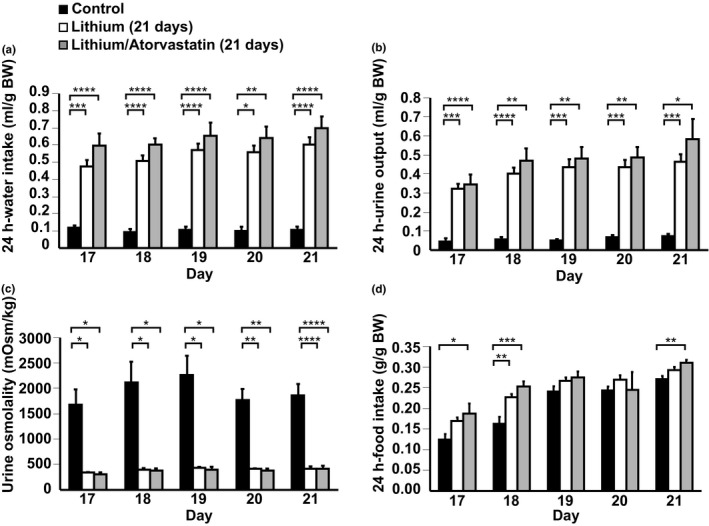
Atorvastatin has no preventive effect on Li‐induced polydipsia, polyuria, and urine hypoosmolality in mice. The last 5 days of treatment, urine was collected in metabolic cages in the prevention experiment. Water intake (panel a), urine output (panel b), urine osmolality (panel c), and food intake (panel d) were measured (*n* = 6 in each group). All results are presented as mean ± SE. *p < 0.05; **p < 0.01; ***p < 0.001; ****p < 0.0001

**FIGURE 6:**
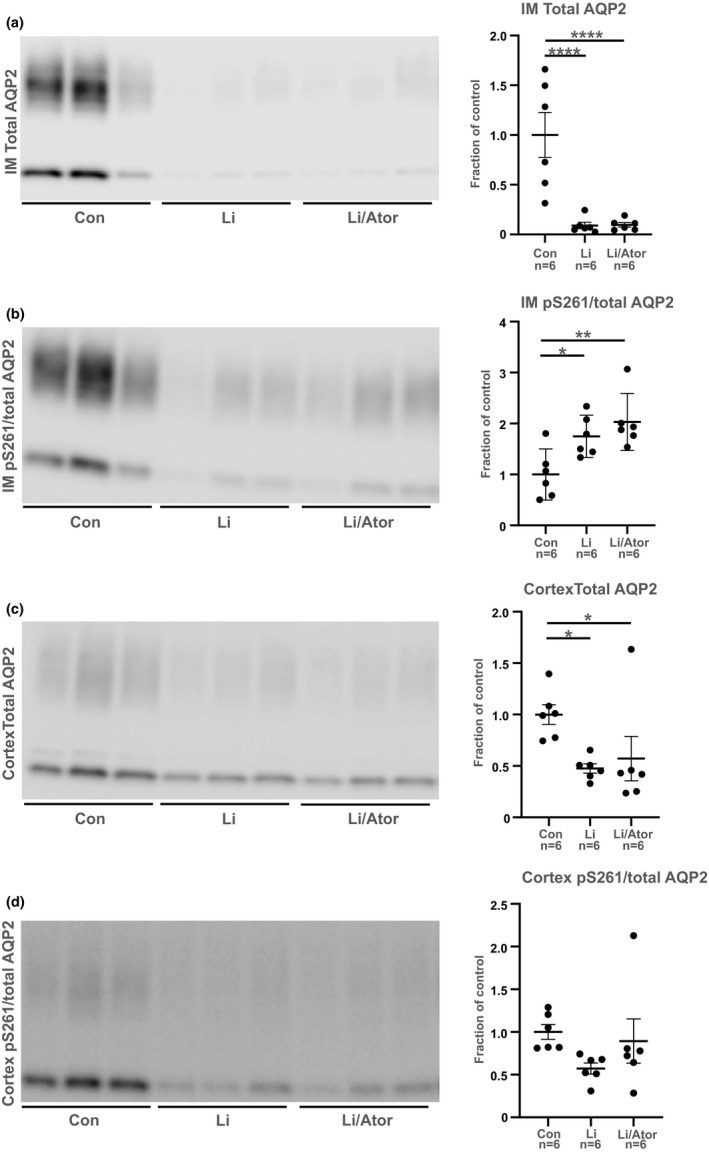
Atorvastatin did not prevent the Li‐induced changes in total AQP2 and S261‐AQP2 expression in renal inner medulla and cortex. Western blots of total AQP2 (panel a and c) and pS261‐AQP2 (panel b and d) in kidney samples from renal medulla (IM, panel a and b) and cortex (panel c and d) from the prevention study are shown. Densitometries are presented as mean ± SE. *p < 0.05; **p < 0.01; ****p < 0.0001

To investigate whether atorvastatin could prevent development of Li‐NDI, mice were challenged either with a control diet or a diet enriched with Li alone or with combined Li/atorvastatin for 3 weeks. There were no significant changes in BW at baseline and at the end of the experiment between the three groups (Table 1). Atorvastatin caused a tendency to decreased serum triglycerides levels compared to controls, although not significant (*p* = 0.0535; Table 1). There were no significant changes in total cholesterol, HDL, and LDL (Table 1). As expected, Li caused a significant increase in water intake and urine output in parallel with a significant decrease in urine osmolality (Figure 5a–c). However, there were no significant changes between the Li and the Li/atorvastatin group (Figure 5a–c). Thus, atorvastatin did not prevent the Li‐induced polyuria and polydipsia. At day 19 and 20, there were no differences in food intake between the three groups (Figure 5d). At day 21, the Li/atorvastatin group had a significantly higher food intake compared to the controls. Western blot revealed that in kidney inner medulla (IM), Li caused a decrease in AQP2 abundance and an increase in the abundance of pS261‐AQP2 (Figure 6a,b). These changes were not attenuated by atorvastatin (Figure 6a,b). In kidney cortex, Li and Li/atorvastatin caused a significant downregulation of total AQP2 compared to controls (Figure 6c). There were no significant changes between the Li and the Li/atorvastatin group. pS261‐AQP2 was not affected by either Li or Li/atorvastatin in kidney cortex (Figure 6d).

**TABLE 1:** Functional data for mice from prevention experiment

	Control	Lithium	Lithium/atorvastatin
BW, g (day 0)	25.8 ± 0.3	26.3 ± 0.4	26.3 ± 0.5
BW, g (day 21)	25.6 ± 0.4	24.5 ± 0.3	24.5 ± 0.2
Serum			
Total cholesterol (mg/dl)	131.5 ± 7.7	133.8 ± 3.4	136.1 ± 4.7
Triglycerides (mg/dl)	132.9 ± 15.3	95.7 ± 6.5	83.3 ± 10.3
High‐density lipoprotein (mg/dl)	42.2 ± 4.9	46.7 ± 2.4	50.5 ± 3.3
Low‐density lipoprotein (mg/dl)	63.0 ± 12.1	68.1 ± 3.0	69.1 ± 1.6

Note

Values are presented as mean ± SE with *n* = 3–6. Serum data are from day 21.

**FIGURE 7:**
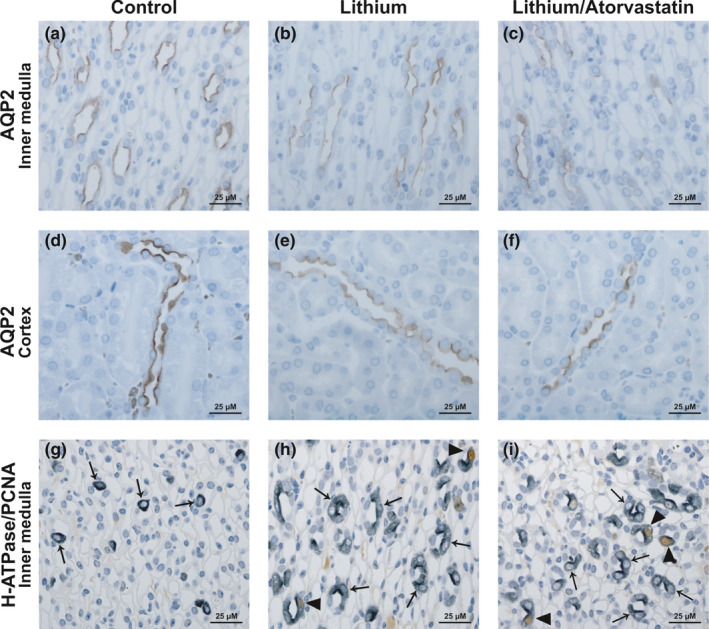
Atorvastatin had no ameliorating effect on the Li‐induced increase in intercalated cells and cell proliferation. Immunohistochemistry on perfusion‐fixed kidney slices from the prevention experiment. Sections were labeled for AQP2 (panels a–f) and double‐labeled for H^+^‐ATPase and PCNA (panels g–i). H^+^‐ATPase‐positive cells are indicated by arrows and H^+^‐ATPase/PCNA‐positive cells are indicated by black triangles

Immunohistochemistry confirmed the Li‐induced downregulation of AQP2 in both IM and cortex in comparison to the control (Figure 7, panels a,b and d,e). Atorvastatin co‐treatment induced an AQP2 expression pattern similar to Li treatment with residual AQP2 expression on the sub‐apical domain of the principal cells (Figure 7, panels c,f). To investigate cellular proliferation and composition of the CD after Li and Li/atorvastatin treatment, we performed double‐labeling with a marker for cellular proliferation (PCNA) and a marker for intercalated cells (B1 subunit of H^+^‐ATPase; Figure 7, panels g–i). In kidney IM, Li induced an increase in the fraction of IC (H^+^‐ATPase‐positive; Figure 7h) and a reduction in the fraction of PC (H^+^‐ATPase‐negative, Figure 7h) compared to control mice (Figure 7g). Co‐treatment with Li/atorvastatin was associated with a similar histological pattern (Figure 7i). Li is well‐known to increase cellular proliferation in the IM of rat kidney (Christensen et al., 2006; Trepiccione et al., 2013). Our data are in line with these previous findings. In the IM, there was an increased amount of both proliferating PC and IC cells (Figure 7h). However, in contrast to rat kidney, the majority of proliferating cells were IC (PCNA and H^+^‐ATPase‐positive, Figure 7h). Co‐treatment with atorvastatin did not affect the Li‐induced proliferation (Figure 7i).

### 3.5 Atorvastatin does not rescue established lithium‐induced NDI in mice

**FIGURE 8:**
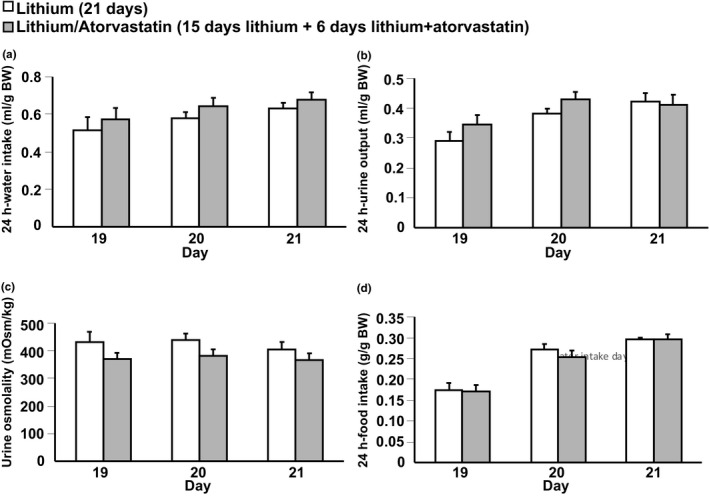
Atorvastatin did not rescue established Li‐induced polydipsia, polyuria, and urine hypoosmolality in mice. Mice were treated with Li for 21 days and the last 6 days of the treatment, the mice also received atorvastatin i.p. (*n* = 10 in each group, rescue study). Water intake, urine output, urine osmolality, and food intake were measured in metabolic cages from day 19 to 21 (panel a–d). All results are presented as mean ± SE

In order to investigate whether atorvastatin could rescue established Li‐NDI, mice were treated with Li for 15 days followed by co‐treatment with Li/atorvastatin the following 6 days. There were no significant changes in BW between the Li and Li/atorvastatin group at baseline (24.8 ± 0.3 vs. 24.3 ± 0.3 g) and at the end of the experiment (23.3 ± 0.3 vs. 22.9 ± 0.4 g). Similar to the prevention experiment, Li caused polydipsia already within the first week of treatment (data not shown). The water intake (measured in regular cages and not corrected for BW) was significant decreased in the Li/atorvastatin group compared to the Li group at day 17 (35.4 ± 1.3 vs. 30.2 ± 0.7 ml, *p* < 0.01) and at day 18 (27.4 ± 1.0 vs. 24.3 ± 1.1 ml, *p* < 0.05). However, there were no significant changes in water intake, urine output, urine osmolality, and food intake between the Li and Li/atorvastatin group at day 19–21 (measured in metabolic cages; Figure 8a–d). Western blotting of samples from IM and cortex revealed no changes in total AQP2 and pS261‐AQP2 abundances between the Li and Li/atorvastatin groups (Table 2). Immunohistochemistry using sections double‐labeled for PCNA and H^+^‐ATPase revealed no changes between the two groups (data not shown).

**TABLE 2:** Western blotting of kidney samples from rescue experiment

	Lithium	Lithium/atorvastatin
Inner medulla		
Total AQP2	1.00 ± 0.18	0.90 ± 0.14
pS261‐AQP2/total AQP2	1.00 ± 0.11	0.98 ± 0.11
Cortex		
Total AQP2	1.00 ± 0.05	1.10 ± 0.14
pS261‐AQP2/total AQP2	1.00 ± 0.12	0.84 ± 0.10

Note

Values are presented as fraction of controls and mean ± SE with *n* = 10.

### 3.6 Atorvastatin does not prevent polyuria induced by potassium depletion in mice

**FIGURE 9:**
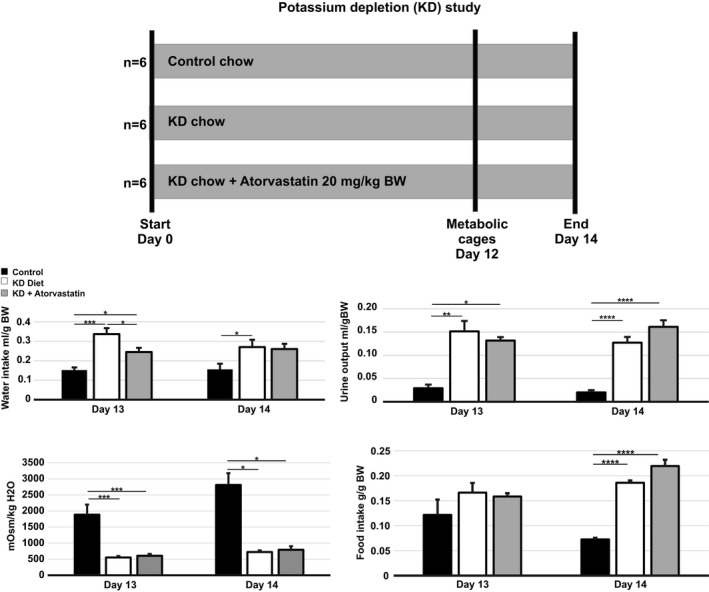
Atorvastatin did not prevent polyuria and urine hypoosmolality in potassium‐depleted mice. Rats were subjected to a potassium‐deficient (KD) diet for 14 days. The last 3 days of the experiment, mice were placed in metabolic cages and water intake, urine output, urine osmolality, and food intake were measured. All results are presented as mean ± SE. *p < 0.05; **p < 0.01; ***p < 0.001

**FIGURE 10:**
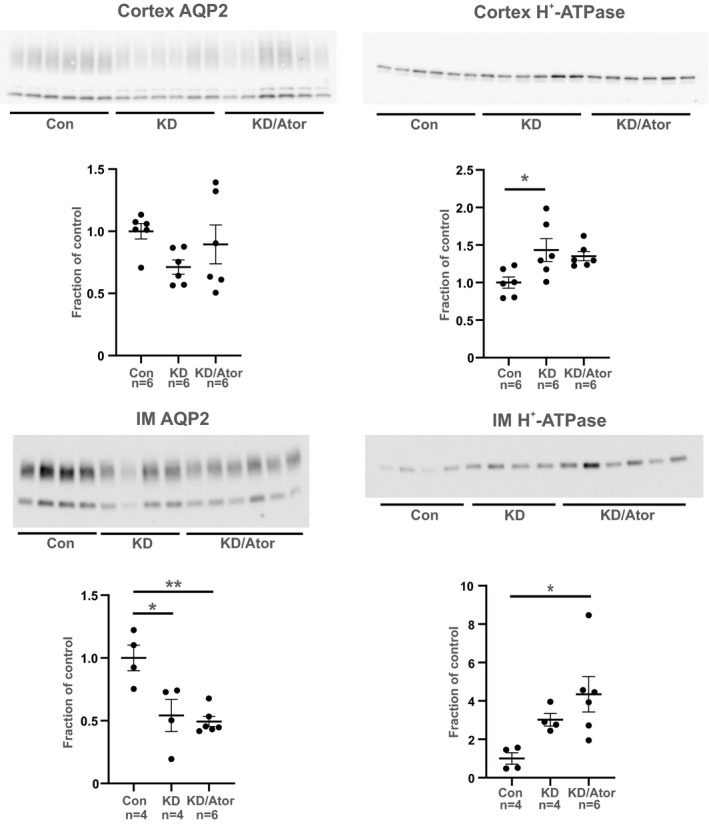
Atorvastatin did not prevent AQP2 downregulation in inner medulla of potassium‐depleted mice. Western blotting of total AQP2 and H^+^‐ATPase in samples from cortex and inner medulla (IM) of potassium‐depleted rats. All results are presented as mean ± SE. *p < 0.05; **p < 0.01

To investigate whether atorvastatin is able to prevent polyuria induced by potassium depletion, mice were treated with a control diet, a KD diet or with combined KD/atorvastatin diet for 14 days (Figure 9). As expected, KD induced a significant reduction in serum potassium levels and in urinary potassium excretion compared to the control group (Table 3). Reduced serum potassium was associated to the development of hypoosmotic polyuria and polydipsia in KD mice compared to mice fed with normal chow (Figure 9). As a consequence, KD mice presented with signs of dehydration as suggested by a significant increase in serum sodium concentration and reduced BW compared to control mice (Table 3). The adjunction of atorvastatin to KD affected the BW, but did not affect the other parameters (Table 3; Figure 9). However, atorvastatin co‐treatment prevented the KD increase in total cholesterol level (Table 3). Finally, no significant changes were observed in serum triglycerides (Table 3). There were no differences in food intake at day 13. Western blot of samples from cortex revealed a tendency, although not statistically significant, to AQP2 downregulation in the KD group compared to controls (Figure 10). The KD group showed a significant upregulation of H^+^‐ATPase protein expression in cortex. In IM, AQP2 was significantly downregulated in the KD and the KD/atorvastatin group compared to controls (Figure 10). The expression of H^+^‐ATPase was upregulated in the KD/atorvastatin group compared to controls (Figure 10).

**TABLE 3:** Functional data for potassium‐depleted mice

	Control	0%potassium	0%potassium/atorvastatin
BW, g (day 0)	22.3 ± 1.03	21.3 ± 0.37	21.1 ± 0.32
BW, g (day 14)	22.4 ± 0.60	20.1 ± 0.34**	21.0 ± 0.32
Urine			
24 h potassium excretion (mmol)	0.69 ± 0.11	0.015 ± 0.002**	0.018 ± 0.003**
24 h sodium excretion (mmol)	0.52 ± 0.08	0.27 ± 0.03*	0.31 ± 0.04*
Serum			
Potassium (mM)	6.79 ± 0.27	4.82 ± 0.33***	5.27 ± 0.26**
Sodium (mM)	157.9 ± 1.7	165.9 ± 1.3**	164.7 ± 1.5*
Total cholesterol (mg/dl)	145.6 ± 5.8	161.7 ± 4.9**	150.8 ± 4.2
Triglycerides (mg/dl)	91.5 ± 6.7	110.7 ± 8.2	106.3 ± 9.7
High‐density lipoprotein (mg/dl)	45.4 ± 1.9	59.7 ± 2.0**	55.9 ± 2.1
Low‐density lipoprotein (mg/dl)	79.3 ± 4.1	81.2 ± 5.2	75.4 ± 5.0

Note

Values are presented as mean ± SE with *n* = 6. Serum and urine data are from day 14.

* *p* < 0.05

** *p* < 0.01

*** *p* < 0.001.

## 4 DISCUSSION

This is the first study to analyze the effect of statins in two acquired forms of NDI (induced by Li treatment or by potassium depletion. Previously, a preliminary cross‐sectional study of 71 Li users has suggested the use of atorvastatin in treatment of Li‐NDI patients; Elie et al., 2015). Recently, we showed, that atorvastatin for 12 weeks (using a relative low dose in order to minimize side effects) did not significantly improve urine osmolality in patients with partial NDI (UOsm < 600 mOsm/kg) and the data suggested potential greater utility of atorvastatin in patients with more severe NDI (UOsm < 300 mOsm/kg; Fotso et al., 2021).

Since acute statin treatment, 1 h simvastatin treatment of LLC‐AQP2 cells or BB rat kidney slices as well as 6 h fluvastatin treatment of normal mice, have shown increased trafficking of AQP2 (Li et al., 2011; Procino et al., 2011), we tested the acute effects of statins in the mCCD_c1l_ cell line. Both atorvastatin, simvastatin, and fluvastatin increased AQP2 trafficking in the cell line confirming that the cell line is suitable for studying the effect of statins on Li‐NDI.

In contrast to the acute effects of statins, we show that atorvastatin is not able to prevent or rescue the Li‐induced polyuria and polydipsia in mice. Atorvastatin had no effect on the Li‐induced AQP2 downregulation and cellular remodeling in the kidney. Furthermore, the Li‐induced AQP2 downregulation was not reversed by atorvastatin or simvastatin in the mCCD_c1l_ cell line. In order to investigate whether atorvastatin was able to prevent another form of acquired NDI, mice were subjected to a KD diet combined with atorvastatin. The KD diet induced a hypoosmotic polyuria associated with lower serum potassium. However, the co‐treatment with atorvastatin was not able to improve the water concentration ability neither the AQP2 abundance. Thus, our results do not support a positive effect of statins on urinary concentration in Li‐ or hypokalemia‐induced NDI.

Statins have been shown to have an effect in other NDI models. These include simvastatin treatment of hypercholesterolemic patients for 12 weeks, which increased AQP2 urine excretion, reduced diuresis, and increased urine osmolality (Procino et al., 2016), and in C57BL/6 mice, fluvastatin treatment for 7 days also reduced urine output and increased urine osmolality (Procino et al., 2011). Furthermore, in animals with X‐linked NDI or central DI a short‐term effect (up to 6 h) of statins on AQP2 membrane accumulation, urine output and osmolality was seen (Li et al., 2011; Procino et al., 2014). In X‐linked NDI, the urinary concentrating defect is due to mutations in the vasopressin V2 receptor and most mutant forms of the receptor are retained in the Golgi apparatus and are not transported to the membrane (Bichet & Bockenhauer, 2016). Central diabetes insipidus (DI) is characterized by vasopressin deficiency (Bernal et al., 2016). Thus, in these two forms of DI, statins likely bypass the vasopressin V2 receptor pathway and exert its function in an otherwise intact cellular system. Similar in normal mice and in hypercholesterolemic patients, statins act on intact kidney cells. Consistently, statins exerts both long‐term and short‐term effects, which are independent of the vasopressin V2 receptor. Long‐term statin treatment may act by inhibiting cholesterol synthesis thereby lowering the content of cholesterol in the plasma membrane leading to a potential reduction in AQP2 endocytosis (Procino et al., 2010). Short‐term statin treatment is suggested to have an effect on cytoskeleton reorganization through downregulation of Rho GTPase activity also leading to decreased AQP2 endocytosis (Bonfrate et al., 2015).

In Li‐NDI, Li enters the principal cells via ENaC (Christensen et al., 2011; Kortenoeven et al., 2009) and it affects multiple cellular pathways, that is, MAP kinases, cell junctions, membrane transport, cytoskeleton, cellular proliferation, cell death, and cell morphology (Nielsen et al., 2008; Trepiccione et al., 2014). In hypokalemia‐induced NDI, AQP2 downregulation is suggested to result from reduction in apical labeling of pS256‐AQP2 and degradation of total AQP2 and p261‐AQP2 in autophagosomes (Kim et al., 2019). In these two models, the downregulation in AQP2 abundance (long‐term AQP2 regulation) may overrule the potential beneficial effect of statins on AQP2 trafficking (short‐term AQP2 regulation). The long‐term effects of statins on AQP2 abundance have not been investigated in X‐linked NDI and central DI (Li et al., 2011; Procino et al., 2014). In our study, we have chosen the durations of statins treatments, so they corresponds to durations of Li treatments previously shown to develop NDI in vivo (3 weeks) and to downregulate AQP2 in vitro (48 h; Christensen et al., 2004, 2006; Kortenoeven et al., 2009; Trepiccione et al., 2013). We also chose the long‐term duration, as the protocol then had more relevance to the clinical situation in human lithium and statin treatment. Although we used a dose of atorvastatin corresponding to statin doses used in previous studies (Dimmeler et al., 2001; Nachtigal et al., 2006; Sparrow et al., 2001) it cannot be excluded either that the dose of atorvastatin has an impact and thus a higher dose of statins is required to get a beneficial effect on urine concentration.

In the Li study, atorvastatin caused a tendency to decrease levels of serum triglycerides, although not significant. Initially, we had expected atorvastatin treatment to lower LDL and total cholesterol. However, treatment with statins in different rabbit, rat and mice strains have shown to have a varied outcome on these parameters (Pecoraro et al., 2014). The study concluded that rabbits were the best model for observing a cholesterol reduction and furthermore, the greatest reduction in cholesterol was seen in animals on a high‐fat diet. Consistent with our study, C57BL/6J mice on normal diet did not lower total cholesterol levels in response to atorvastatin, simvastatin, or pitavastatin treatment (Aprahamian et al., 2006; Nachtigal et al., 2006; Yagi et al., 2008). In humans, statins have also been shown to be the most effective in reducing mortality when cholesterol levels are above well‐defined limits, which occurs when humans live on a high‐fat western diet. The lithium serum levels were not measured in the present studies, but we have used a protocol previously shown to result in serum levels in the physiological range (Christensen et al., 2011). We can assume that the mice have consumed the same amount of lithium, since there were no differences in food intake between the experimental groups. Suprisingly, we could see an increase of total cholesterol in KD mice. This could be due to the relative dehydation status of the KD mice, but a primary effect of the KD cannot be excluded. Co‐treatment with atorvastatin was able to prevent this effect on cholesterol.

In conclusion, our study shows that atorvastatin does not appear to be able to prevent or rescue Li‐NDI in mice. Furthermore, atorvastatin did not prevent NDI induced by potassium depletion in mice.

## CONFLICT OF INTEREST

Soham Rej received an investigator‐initiated grant from Satellite Healthcare for another project.

## AUTHOR CONTRIBUTION

Maria L. Thomsen planned and conducted experiments, analyzed the results, prepared figures, and participated in writing the manuscript. Camilla Grønkjær planned and conducted experiments, and participated in writing the paper. Anna Iervolino conducted experiments and participated in writing the manuscript. Soham Rej participated in planning experiments, analyzing results, and writing the paper. Francesco Trepiccione planned experiments, analyzed results, and participated in writing the manuscript. Birgitte M. Christensen planned and conducted experiments, analyzed the results, prepared figures, and participated in writing the paper.
